# Analysis of dietary patterns and nutritional adequacy in lactating women: a multicentre European cohort (ATLAS study)

**DOI:** 10.1017/jns.2021.7

**Published:** 2021-03-11

**Authors:** Dantong Wang, Frank Thielecke, Mathilde Fleith, Myriam C. Afeiche, Carlos A. De Castro, Cecilia Martínez-Costa, Kirsti Haaland, Giovanna Marchini, Massimo Agosti, Magnus Domellöf, Maria Jose Costeira, Claude Billeaud, Mireille Vanapee, Jean-Charles Picaud, Tinu Mary Samuel

**Affiliations:** 1 Nestlé Research, Vers-chez-les-Blanc, P.O. Box 44, 1000 Lausanne 26, Switzerland; 2 Swiss Distance University of Applied Sciences, Regensdorf-Zurich, Switzerland; 3 T2 Bene Ltd, Bettenstrasse 60a, 4123 Allschwil, Switzerland; 4 Hospital Clínico Universitario, University of Valencia, Valencia, Spain; 5 Oslo University Hospital, 0010 Oslo, Norway; 6 Karolinska University Hospital, Stockholm, Sweden; 7 Ospedale del Ponte, Varese, Italy; 8 Department of Clinical Sciences, Umeå University, SE90185 Umeå, Sweden; 9 Instituto de Investigação em Ciências da Vida e Saúde, Braga, Portugal; 10 Hôpital des enfants, CHU Pellegrin, Bordeaux, France; 11 Astrid Lindgren Children's Hospital, Karolinska University Hospital/Karolinska Institutet, Stockholm, Sweden; 12 Hôpital de la Croix Rousse, Lyon, France

**Keywords:** Nutrition adequacy, Lactating mothers, Multicentre European cohort, Cluster analysis

## Abstract

Eating habits of lactating women can influence the nutrient composition of human milk, which in turn influences nutrient intake of breastfed infants. The aim of the present study was to identify food patterns and nutritional adequacy among lactating women in Europe. Data from a multicentre European longitudinal cohort (ATLAS study) were analysed to identify dietary patterns using cluster analysis. Dietary information from 180 lactating women was obtained using 3-d food diaries over the first 4 months of lactation. Four dietary patterns were identified: ‘vege-oils’, ‘fish-poultry’, ‘confectionery-salads’ and ‘mixed dishes’. Nutrition adequacy was not significantly different between clusters, but the ‘vege-oils’ cluster tended to yield the highest nutrition adequacy measured by Mean Adequacy Ratio. Compared with European dietary reference values (DRVs) for lactating women, women in all clusters had inadequate intakes of energy, pantothenic acid, folate, vitamin C, vitamin A, vitamin D, zinc, iodine, potassium and linoleic acid. Adequate intake for fibre and α-linolenic acid was only achieved in the ‘vege-oils’ cluster. Overall, fat intake was above DRVs. The present study showed that various dietary patterns do not adequately supply all nutrients, indicating a need to promote overall healthy dietary habits for European lactating women.

## Introduction

Lactation raises the nutrient needs of mothers, mainly because of nutrient transfer through breastmilk^(1)^. Yet, inadequate nutrient intake is frequent in women during lactation because women often do not change their dietary habits from pre-conception to postpartum periods^(2,3,4)^. Further dietary concerns include the use of weight-loss diets to return to pre-pregnancy weight, suboptimal intake of selected nutrients because of health conditions or poor food choices, not having time to eat properly and/or excessive use of caffeine and alcohol. Inadequate nutrient intake during lactation matters because it increases the risk of deficiency for selected nutrients; nutrient deficiency and/or prolonged inadequate caloric intake have been linked with low volume and inadequate levels of nutrients in human milk (HM)^(5,6,7)^. International and national health authorities worldwide, including the European Food Safety Authority (EFSA)^(8)^, have thus established specific nutrient requirements for lactating women.

Nutrient inadequacies in lactating mothers have been reported from different regions of the world^(2,3,4)^. A recent analysis in Spanish women concluded that diet quality between immediate postpartum (up to 40 d after delivery) and during pregnancy did not differ regarding adherence to the Mediterranean diet. However, there was a trend of lower consumption of foods associated with a healthy diet^(9)^. This trend was also observed in lactating women at 6 months’ postpartum with a decrease in the intake of cereals, vegetables, fruits and dairy products^(10)^. Copp *et al.* found that mean maternal dietary intake for vitamin A, vitamin D, choline and docosahexaenoic acid (DHA) did not meet US dietary reference intakes or expert recommendations^(11)^. For example, 58 % women met the current recommendations for vitamin A, 44 % for vitamin D, 58 % for choline and only 5 % for DHA. Inadequate nutrient intakes were also reported from a sample of lactating women within 90 d postpartum in China^(12)^. The authors reported that mean daily energy levels fell 11–17 % below Chinese estimated energy requirements, while fat intake was 9–77 % above the recommended nutrient intake (RNI). Furthermore, the intake of vitamin C, folate and dietary fibre was below respective RNIs. Low adequacy of micronutrient intake was also reported among rural Indonesian lactating women, where the overall mean population prevalence of micronutrient adequacy was only 57 %^(13)^. Suboptimal nutrient intake in pregnant and lactating women has also been reported from Spain^(3)^, the UK ^(2)^ and Norway^(14)^.

In nutritional epidemiology, dietary patterns are commonly used as a method to assess habitual diet as overall dietary exposure^(15,16)^. Evaluation of dietary exposure on a holistic basis offers certain advantages over looking at nutrients in isolation, e.g. combinations of nutrients that are likely to be interactive or synergistic, or correlations of nutrients which make it difficult to delineate effects of individual nutrients and cumulative effects of multiple nutrients that may be larger than single nutrients^(17)^.

The purpose of this analysis was to identify dietary patterns using cluster analysis in a multicentre longitudinal prospective cohort and assess the nutrition adequacy of the identified patterns. To our knowledge, this is the first cluster analysis of a European multicentre prospective longitudinal cohort to assess dietary patterns and nutrition adequacy among lactating mothers.

## Methods

### Study population

The data used for this analysis are part of the ATLAS study, a European multicentre, longitudinal, observational, exploratory cohort designed to characterise HM and its association with maternal and infant parameters^(18)^. Healthy lactating mothers from seven European countries (thirteen centres) were invited for the present study: France (three centres), Italy, Norway, Portugal (three centres), Romania (two centres), Spain and Sweden (two centres). Ethical approval was obtained from local authorities. The ATLAS study was registered at www.ClincalTrials.gov (NCT01894893). Participants were eligible if they were between 18 and 40 years old at the time of enrolment, had a body mass index (BMI) before pregnancy between 19 and 29, had decided to exclusively breast-feed their newborns from birth up to 4 months of age, and had signed the Informed Consent Form.

### Assessment of dietary intake and dietary adequacy

Food diaries were collected at six time points throughout postpartum and up to 4 months of lactation. The participants were observed immediately preceding delivery (V0) and then observed for 6 follow-up visits after delivery, i.e. at day 2 (V1), 17 (V2), 30 (V3), 60 (V4), 90 (V5) and 120 (V6). At each visit, participants were asked to provide information on their dietary consumption over the preceding 3 d. Dietary intake was estimated using 3-d food diaries and calculated as a 3-d average. Participants’ dietary information was then translated into consumption data, food group data and nutrient daily intake using the software Nutrilog and the French food composition table (CIQUAL). Food group classification was assigned according to the 2013 version of CIQUAL and nutrient composition using the 2017 version^(19)^. Nutrition adequacy was assessed by comparing the relative intake with Average Requirements (ARs) proposed by the EFSA^(8)^. For nutrients without AR, Adequate Intake was used; for total fat and carbohydrates, the lower range of Reference Intake ranges for macronutrients (RI) were used. Furthermore, for each food pattern, the Mean Adequacy Ratio (MAR) was calculated as an overall measure of nutrient adequacy^(20,21)^. The MAR was based on the Nutrient Adequacy Ratio (NAR) calculated for twenty-eight nutrients including energy. NAR is a measure that expresses an individual's intake of a nutrient as a percentage (capped at 100 %) of the corresponding recommended allowance for that nutrient, which was EFSA's Population Reference Intakes (PRIs), given the respondent's age and sex. The MAR is then calculated by averaging the NAR of each nutrient. The MAR allows to estimate overall nutritional adequacy of a given cluster, rather than focusing on specific nutrients that may not alone indicate healthy diet composition.

### Statistical analysis

The K-means clustering method, which is commonly used in dietary pattern analysis^(22)^, was applied to identify dietary patterns from the early stage and up to 4 months of lactation. Analyses were performed on the twenty-nine food group variables for participants (*n* 180) in the V2–V6 subset. V1 data were excluded because women were usually still in the hospital at this timepoint, which is not a reflection of the normal dietary habits. The ideal number of clusters K was determined by following the gap statistic^(23)^.

Analyses were performed for 3–5 clusters. The four-cluster solution was chosen using the approach described earlier, basically taking into consideration the amount of variation explained by the solution, size and interpretability of each cluster and the stability of the solution, which was evaluated using linear discriminant analysis. Twenty-nine main food groups from the CIQUAL database 2013 were considered for the analyses: ‘Fruits’, ‘Legumes’, ‘Starchy tubers’, ‘Nuts and seeds’, ‘Breads and rolls’, ‘Breakfast cereals and cereal bar’, ‘Cereals and pasta’, ‘Pastry and biscuits’, ‘Eggs and egg products’, ‘Milk and milk products’, ‘Cheeses’, ‘Mixed salads’, ‘Mixed dishes’, ‘Sandwiches’, ‘Fish’, ‘Shellfish and mollusc’, ‘Meat products’, ‘Poultry’, ‘Red meat’, ‘Coffee tea cocoa beverages’, ‘Soft drinks’, ‘Juices and nectars’, ‘Fats and oils’, ‘Seasonings and sauces’, ‘Soups and stocks’, ‘Baby food’, ‘Dietary Supplements’, ‘Offals’ and ‘Products for special nutritional use’. The latter four were excluded because either only a small number of women consumed significant amounts, and/or they were not considered as food (i.e. dietary supplements, as reported by five participants). Supplementary Table S4 of Supplementary material details the specific food groups and the food items. Food intake was recorded 3 d before each visit. For each recorded day, intake of fifty-four nutrients for each volunteer were calculated using food item record and food composition values of the CIQUAL 2017 database. To create the dataset for further analyses of dietary patterns, participants with outlier diets (less than 1074⋅8 kcal or more than 4776⋅9 kcal) were removed. A similar approach has been described elsewhere^(24)^. Insufficiently robust dietary information (i.e. fewer than two non-outlier diets reported for a participant across the 3-d survey period) led to the removal of the participant, too.

Statistical analyses were performed with SAS version 9.3 (SAS Institute, Cary NC, USA) and R version 3.2.3. One-way analysis of variance (ANOVA) on ranks and Fisher's exact tests were used to verify differences between clusters. Data are presented as mean ± sd unless stated otherwise.

## Results

In total, 197 participants with dietary intake were initially included in this analysis. The removal of outliers and lactating mothers with only V1 dietary intake resulted in a final sample size of 180 healthy lactating mothers from eight centres in six European countries, France and Portugal each two centres, Italy, Norway, Spain and Sweden each one centre being included in the analysis. Data from all countries were combined and analysed as one ‘European’ dataset.

Four distinct clusters were identified. The clusters were labelled according to the top food groups consumed of each cluster, i.e. vege-oils, fish-poultry, confectionery-salads and mixed dishes. The ‘vege-oils’ included 40 (22⋅2 %) mothers, ‘fish-poultry’ 57 (31⋅7 %) mothers, ‘confectionery-salads’ 38 (21⋅1 %) mothers and ‘mixed dishes’ 45 (25 %) mothers. Overall, significant differences between clusters were observed for parity (*P* = 0⋅0004), mode of delivery (*P* = 0⋅0004) and infant sex (*P* = 0⋅0296). See Table 1 for the total cohort and clusters characteristics. A comparison of clusters revealed that the ‘fish-poultry’ cluster included significantly more mothers having had C-sections than the other clusters. Significant differences were also observed regarding parity, where cluster ‘confectionery-salads’ showed fewer first born compared with clusters ‘vege-oils’, ‘fish-poultry’ and ‘mixed dishes’. With respect to the infants’ sex, cluster ‘mixed dishes’ included significantly more females than the other three clusters (Table 1).

**Table 1.** Characteristics of lactating mothers by total population and by clusters

	Total population	Vege-oils	Fish-poultry	Confectionery-salads	Mixed dishes
*N* (%)	180 (100)	40 (22⋅2)	57 (31⋅7)	38 (21⋅1)	45 (25)
Age (years)	31⋅5 (3⋅9)	32⋅0 (3⋅7)^a^	31⋅2 (3⋅6)^a^	31⋅5 (4⋅4)^a^	31⋅4 (4⋅1)^a^
Pre-pregnancy BMI (kg/m^2^)	22⋅5 (2⋅6)	22⋅5 (2⋅5)^a^^,^^b^	23⋅3 (2⋅4)^a^	21⋅4 (1⋅8)^b^	22⋅6 (2⋅9)^a^^,^^b^
BMI at 4 months postpartum (V6) (kg/m^2^)	23⋅6 (3⋅1)	23⋅4 (3⋅0)^a^^,^^b^	24⋅9 (3⋅2)^a^	21⋅8 (1⋅8)^b^	23⋅7 (3⋅4)^a^^,^^b^
Gestational age at birth (weeks)	39⋅5 (1⋅2)	39⋅9 (1⋅1)^a^	39⋅1 (1⋅3)^a^	39⋅8 (0⋅9)^a^	39⋅6 (1⋅1)^a^
*Parity* (*N*)					
Primiparous/multiparous	126/54	32/8^a^	47/10^a^	17/21^b^	30/15^a^
*Mode of delivery* (*N*)					
Vaginal/C-section	152/28	37/3^a^	39/18^b^	37/1^a^	39/6^a^
*Infant sex* (*N*)					
Male/female	94/86	22/18^a^	35/22^a^	22/16^a^	15/30^b^

Age, BMI and gestational age at birth expressed as means (sd).

^a,b^ Significant differences between clusters at *P* < 0⋅05 as calculated by Fishers's exact test.

**Fig. 1.**
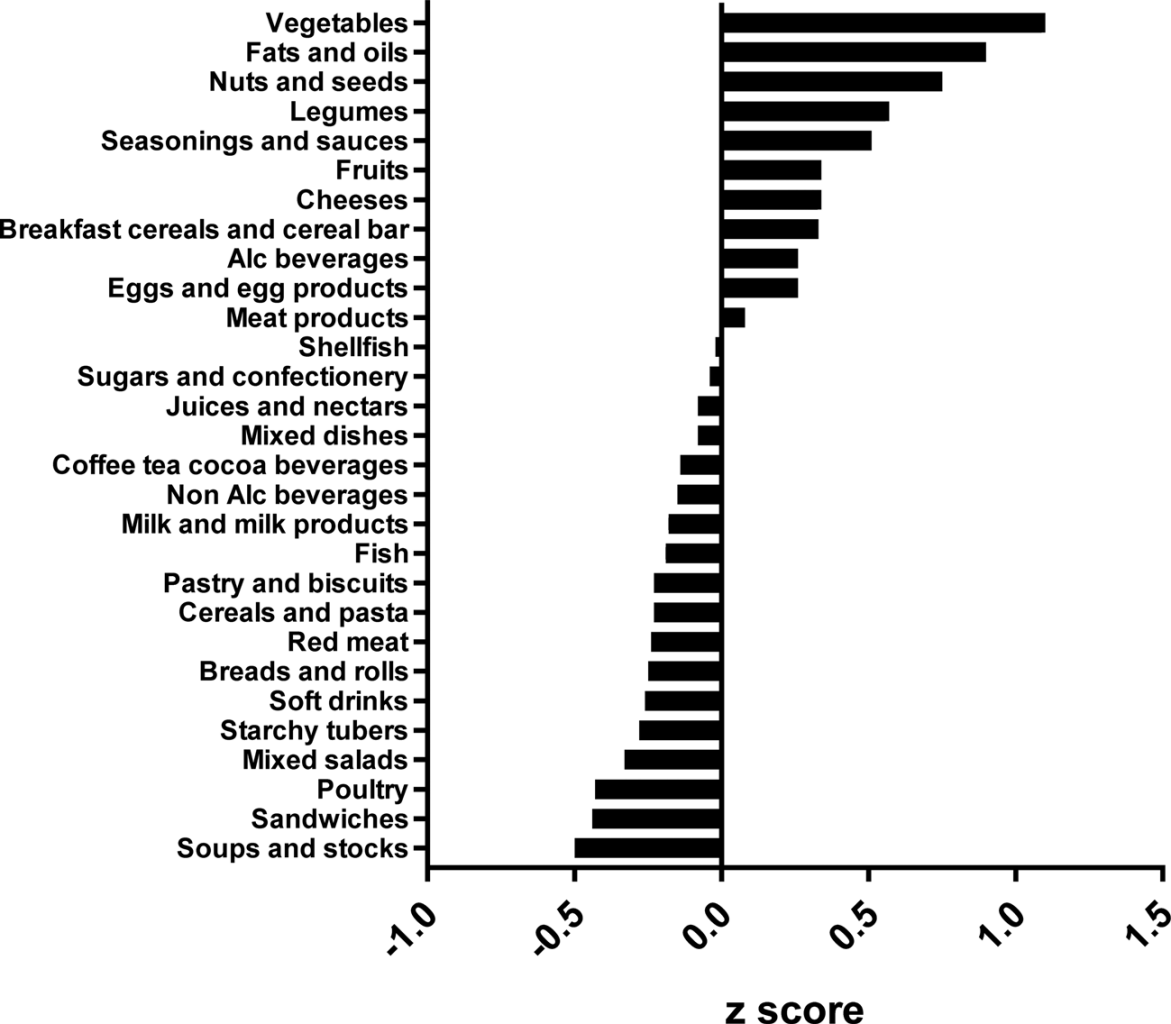
Normalised food intake in cluster ‘vege-oils’.

**Fig. 2.**
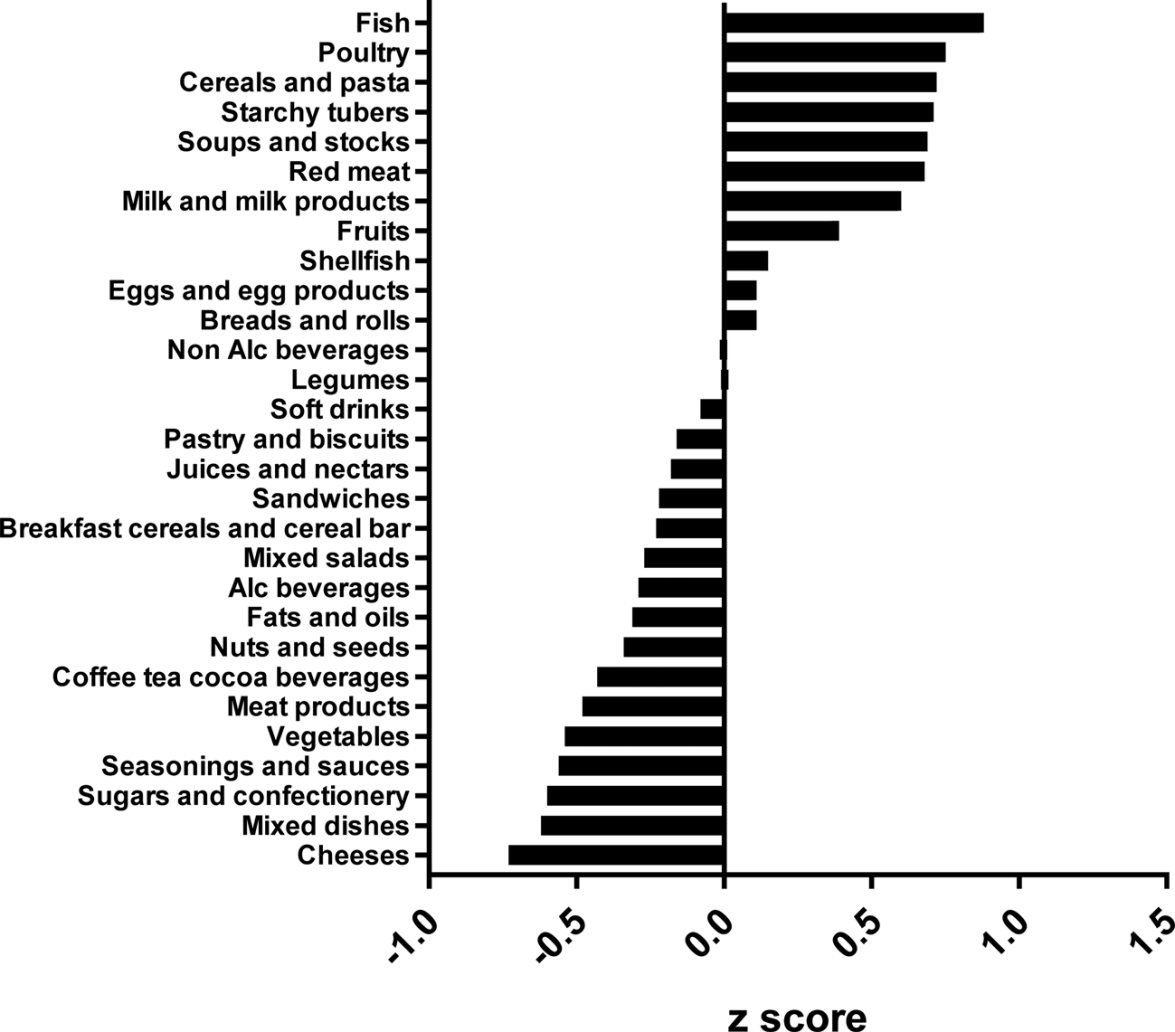
Normalised food intake in cluster ‘fish-poultry’.

**Fig. 3.**
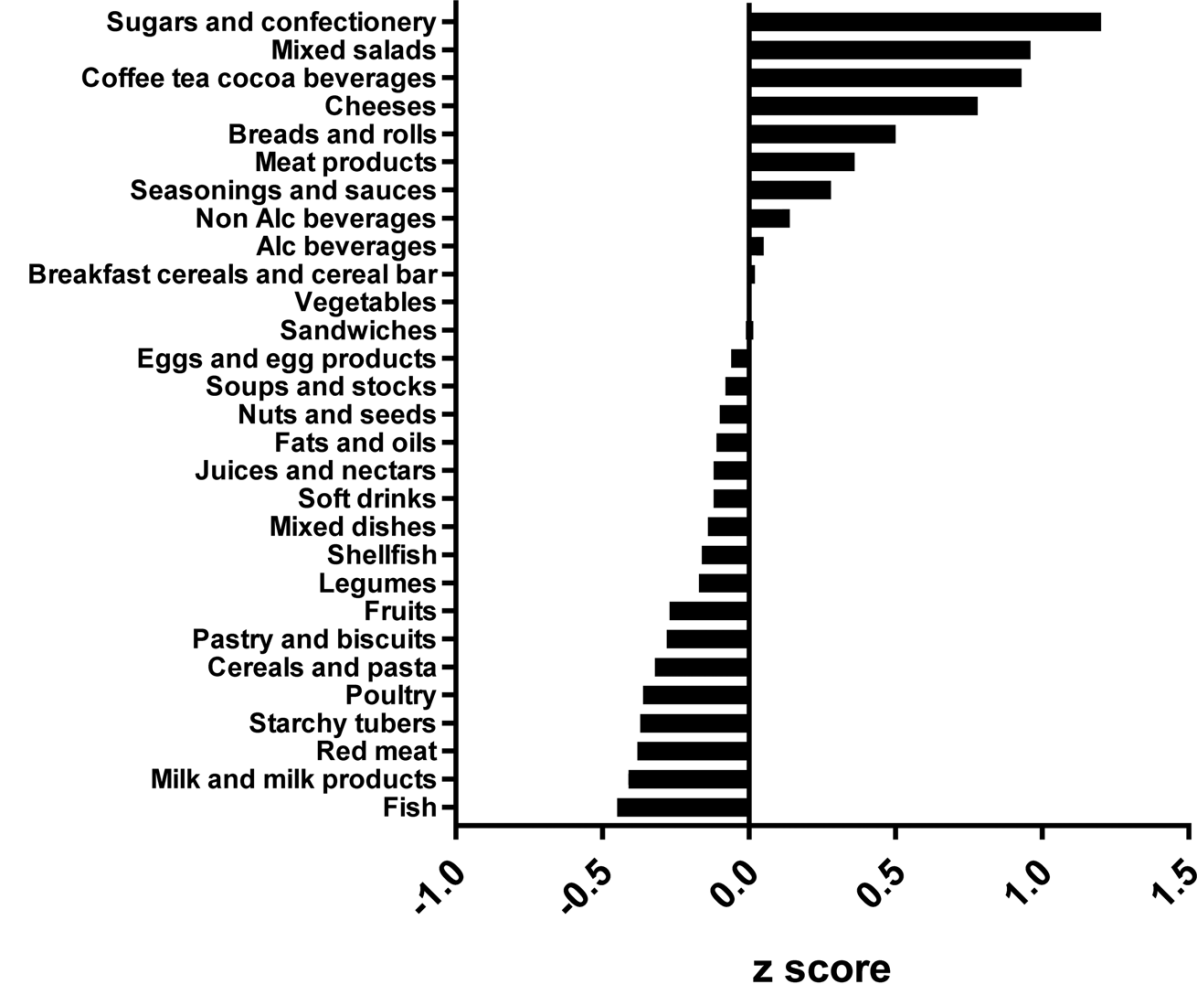
Normalised food intake in cluster ‘confectionery-salads’.

**Fig. 4.**
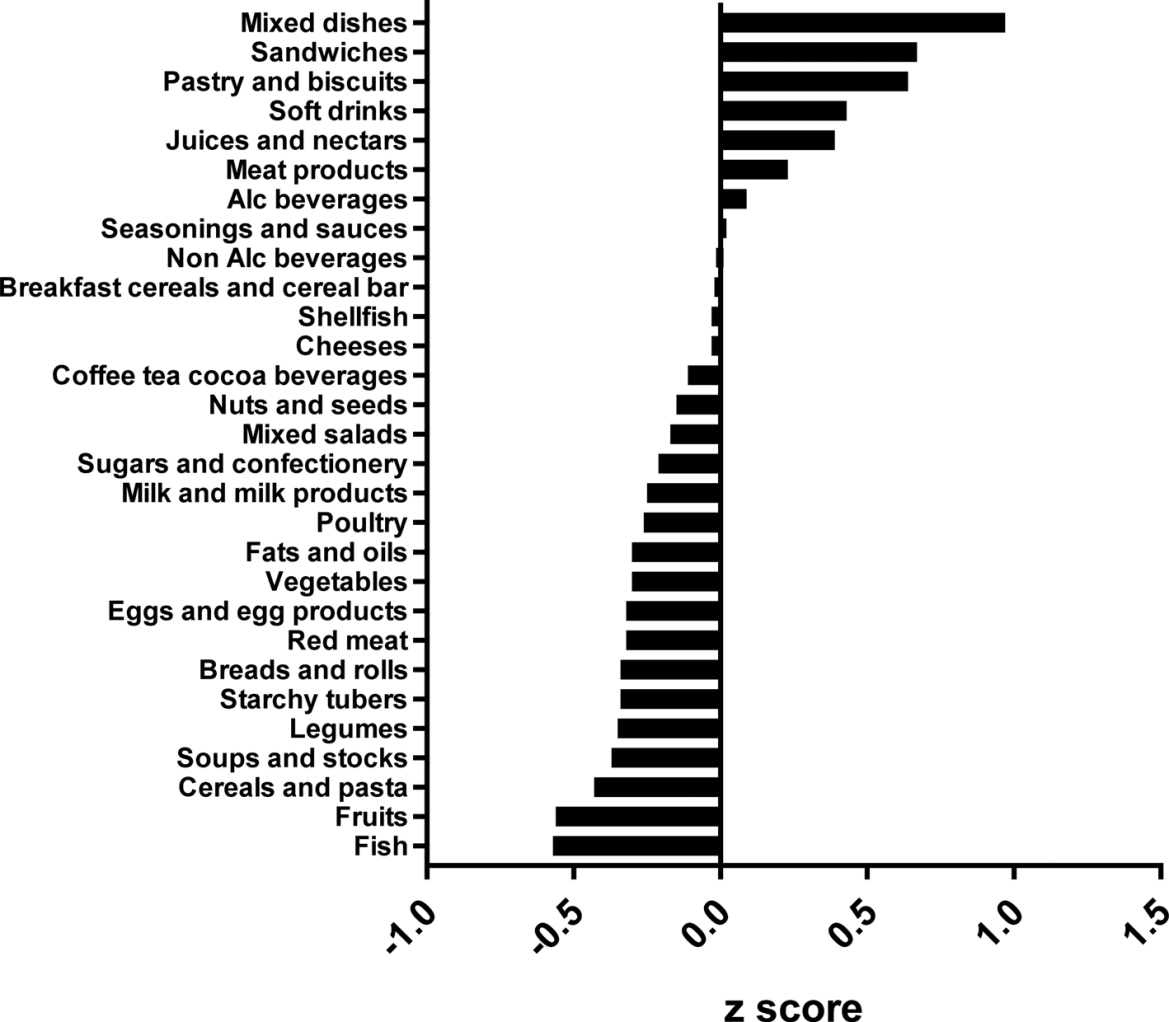
Normalised food intake in cluster ‘mixed dishes’. The blue line represents the 100 % DRV of each nutrient. SFA, saturated fatty acid.

The types of food groups consumed per cluster are illustrated in Figs. 1–4. Women in the ‘vege-oils’ cluster consumed relatively high amounts of ‘vegetables’, ‘fats and oils’, ‘nuts and seeds’, but low amounts of ‘soups and stocks’, ‘mixed salads’ and ‘sandwiches’ compared with the other three clusters (Fig. 1). The consumption of ‘fish’, ‘poultry’, ‘cereals and pasta’ was high in the ‘fish-poultry’ cluster but low amounts of ‘cheeses’, ‘mixed dishes’, ‘sugars and confectionery’ were consumed compared with the other clusters (Fig. 2). Women in the ‘confectionery-salads’ cluster had a relatively high consumption of the food groups such as ‘sugars and confectionery’, ‘mixed salads’ and ‘caffeinated beverages’ but low amounts of ‘fish’, ‘milk and milk products’ as well as ‘red meat’ (Fig. 3). Women in the cluster ‘mixed dishes’ consumed high amounts of ‘mixed dishes’, ‘sandwiches’, ‘pastry and biscuits’ but low amounts of ‘fish’, ‘fruits’ and ‘cereals and pasta’ (Fig. 4).

The mean daily nutrient intake per cluster is presented in Table 2. While daily energy consumption was overall low, with a total population mean of 2004 kcal compared with recommended 2317 kcal^(8)^, it did not differ significantly between clusters, even though specific nutrients varied considerably. Unsurprisingly, the mean consumption of protein was, with 97 g, the highest in the ‘fish-poultry’ cluster compared with all other clusters. In contrast, the mean consumption of fat (60 g), and in consequence thereof saturated fatty acids (SFAs), was lowest in the ‘fish-poultry’ cluster with 24 g. The mean fibre intake was highest in the ‘vege-oils’ cluster (25 g). The mean intake of sodium was highest in the ‘mixed dishes’ cluster (3255⋅5 mg). Total sugar intake did not differ between clusters.

**Table 2.** Raw mean nutrient intake per cluster

	Vege-oils		Fish-poultry		Confectionery-salads		Mixed dishes		Total population		
	Mean	sd	Mean	sd	Mean	sd	Mean	sd	Mean	sd	*P* value
Energy (kcal)	2113	441	1888	331	2115	400	2122	293	2044	377	0⋅209
Protein (g)*	83	19	97	15	81	16	83	13	87	17	0⋅001
Total fat (g)	86	21	60	13	88	20	86	15	78	21	0⋅005
Carbohydrates (g)	237	59	228	47	234	52	242	83	235	49	0⋅339
SFA (g)*	33	9	24	6	38	11	36	9	32	10	<0⋅001
Linoleic acid (g)	8⋅1	3⋅3	4⋅6	1⋅3	6⋅8	2⋅4	6⋅8	1⋅9	6⋅4	2⋅6	0⋅301
α-linolenic acid (g)	1⋅3	0⋅8	0⋅7	0⋅2	0⋅9	0⋅3	1⋅0	0⋅3	1⋅0	0⋅5	0⋅018
EPA (g)	0⋅20	0⋅10	0⋅20	0⋅20	0⋅20	0⋅20	0⋅10	0⋅10	0⋅17	0⋅15	0⋅023
DHA (g)	0⋅20	0⋅20	0⋅40	0⋅20	0⋅20	0⋅20	0⋅20	0⋅20	0⋅25	0⋅22	0⋅004
Fibres (g)*	25	9	18	5	20	5	19	5	20⋅2	6⋅5	<0⋅001
Vitamin D (μg)	6⋅2	8⋅9	5⋅0	1⋅8	5⋅6	9⋅2	4⋅5	4⋅5	5⋅3	6⋅4	0⋅180
Vitamin B12 (μg)	5⋅9	3⋅2	7⋅7	4⋅1	5⋅8	4⋅2	6⋅9	7⋅2	6⋅7	4⋅9	0⋅782
Vitamin B6 (mg)	2⋅2	1⋅4	2⋅0	1⋅4	2⋅0	1⋅5	1⋅8	0⋅6	2⋅0	1⋅0	0⋅027
Folate (μg)	446⋅2	434⋅2	257	62⋅9	378⋅4	435⋅5	306⋅9	89⋅9	337⋅2	298⋅0	0⋅067
Thiamine (mg)	2⋅1	2⋅4	1⋅3	0⋅3	1⋅5	1⋅5	1⋅5	0⋅6	1⋅6	1⋅4	0⋅044
Riboflavin (mg)	2⋅0	1⋅7	1⋅7	0⋅4	2⋅0	1⋅6	1⋅8	0⋅6	1⋅9	1⋅2	0⋅279
Niacin (mg)	21⋅9	16⋅8	23⋅0	4⋅7	20⋅1	17⋅1	19⋅5	6⋅3	21⋅3	11⋅9	0⋅049
Retinol (μg)	398⋅4	287⋅5	247⋅7	96⋅5	496⋅0	324⋅0	494⋅0	463⋅9	395⋅2	327⋅2	0⋅007
β-carotene (μg)*	3691⋅2	2285⋅9	2657⋅0	1507⋅7	3080⋅9	2224⋅1	2094⋅7	1009⋅8	2835⋅8	1856⋅7	<0⋅001
Vitamin A Retinol equivalents (μg)	940⋅2	453⋅8	684⋅3	266⋅1	930⋅3	358⋅0	788⋅0	435⋅8	819⋅0	390⋅1	0⋅672
Vitamin C (mg)	134⋅4	115⋅7	108⋅3	49⋅3	126⋅7	132⋅3	118⋅4	69⋅0	120⋅5	92⋅5	0⋅367
Iron (mg)	16⋅3	18⋅1	8⋅6	1⋅9	13⋅7	12⋅3	12⋅3	8⋅4	16⋅4	56⋅4	0⋅814
Sodium (mg)*	2601⋅1	652⋅9	2239⋅4	585⋅0	2829⋅6	559⋅5	3255⋅5	1668⋅3	2698⋅4	1048⋅7	<0⋅001
Magnesium (mg*)	380⋅0	154⋅0	288⋅9	59⋅3	341⋅4	144⋅1	298⋅6	67⋅8	322⋅6	114⋅1	0⋅001
Calcium (mg)	1077⋅3	416⋅1	813⋅0	287⋅9	1036⋅7	419⋅8	967⋅1	310⋅4	957⋅5	367⋅4	0⋅351
Salt (g)	3⋅9	1⋅6	3⋅9	1⋅2	3⋅6	1⋅1	3⋅7	1⋅4	3⋅8	1⋅4	0⋅055
Zinc (mg)	11⋅5	8⋅1	9⋅3	1⋅8	10⋅9	7⋅9	9⋅7	2⋅2	10⋅2	5⋅5	0⋅115
Potassium (mg)*	3373⋅6	1345⋅6	3088⋅3	610⋅8	2852⋅5	652⋅2	2852⋅4	723⋅2	3043⋅0	877⋅3	<0⋅001
Iodine (mg)	168⋅6	160⋅2	160⋅4	37⋅5	159⋅2	152⋅3	138⋅5	51⋅1	156⋅5	107⋅7	0⋅042
Alcohol (g)	1⋅2	2⋅0	0⋅5	1⋅1	0⋅1⋅4	2⋅1	1⋅3	2⋅5	1	2	0⋅205
Total sugars (g)	95⋅7	25⋅0	90⋅5	29⋅0	97⋅5	27⋅5	98⋅2	24⋅7	95⋅1	26⋅8	0⋅808
MAR	0⋅76	0⋅12	0⋅70	0⋅10	0⋅72	0⋅14	0⋅72	0⋅12	0⋅72	0⋅12	0⋅1

Mean intake was assessed using 3-d food diaries and calculated as a 3-d average. One-way ANOVA on ranks was used. *P < 0⋅05 after Bonferroni correction for multiple comparisons.

Energy adjusted median nutrient intakes per cluster are presented in Table 3. Women in the ‘fish-poultry’ cluster consumed significantly less energy compared with the other three clusters, but had the highest protein and lowest fat intake (*P* < 0⋅05). Relative to energy intake, the ‘vege-oils’ cluster showed the highest intake of fibre, folate and magnesium (*P* < 0⋅05). The ‘mixed dishes’ cluster was characterised by the lowest intake of fibre, β-carotene and zinc (*P* < 0⋅05). Women in the cluster ‘confectionery-salads’ consumed the highest amounts of alcohol and the least amount of thiamine (*P* < 0⋅05).

**Table 3.** Energy adjusted median intakes of nutrients per 1000 kcal among lactating mothers in ATLAS, a European cohort study

	Vege-oils	Fish-poultry	Confectionery-salads	Mixed dishes
Energy (kcal/d)	2060^a^	1827^b^	2102^a^	2106^a^
Protein (g/1000 kcal)	39^a^	52^b^	39^a^	40^a^
Total fat (g/1000 kcal)	40^a^	31^b^	42^a^	40^a^
Carbohydrates (g/1000 kcal)	113^a^	121^b^	110^a,b^	113^a,b^
SFA (g/1000 kcal)	15^a^	12⋅3^b^	18⋅1^c^	17⋅2^c,d^
Linoleic acid (g/1000 kcal)	3⋅4^a^	2⋅3^b^	3⋅1^c^	3⋅1^c,d^
α-linolenic acid (g/1000 kcal)	0⋅50^a^	0⋅35^b^	0⋅43^a,c^	0⋅43^a,c^
EPA (g/1000 kcal)	0⋅06^a^	0⋅10^b^	0⋅05^a^	0⋅03^a^
DHA (g/1000 kcal)	0⋅10^a^	0⋅16^b^	0⋅06^c^	0⋅05^c,d^
Fibres (g/1000 kcal)	11⋅6^a^	9⋅4^b^	9⋅5^b,c^	8⋅4^d^
Vitamin D (μg/1000 kcal)	1⋅9^a^	2⋅6^b^	1⋅5^a^	1⋅5^a^
Vit B12 (μg/1000 kcal)	2⋅5^a^	3⋅5^b^	2⋅2^a^	2⋅3^a^
Vit B6 (mg/1000 kcal)	0⋅90^a^	1⋅06^b^	0⋅84^a,c^	0⋅83^a,c^
Folate (μg/1000 kcal)	168^a^	138^b^	147^b,c^	139^b,c,d^
Thiamine (mg/1000 kcal)	0⋅69^a^	0⋅71^a^	0⋅61^b^	0⋅65^a^
Riboflavin (mg/1000 kcal)	0⋅79^a^	0⋅90^b^	0⋅85^a,b^	0⋅76^a^
Niacin (mg/1000 kcal)	9⋅26^a^	12⋅37^b^	8⋅57^a^	9⋅41^a^
Retinol (μg) /1000 kcal)	148^a^	122^b^	185^c^	174^a,c^
β-carotene (μg/1000 kcal)	1516^a^	1268^a^	1350^a^	1051^b^
Vitamin A Retinol equivalents (μg/1000 kcal)	418^a^	338^b^	421^a^	363^b^
Vitamin C (mg/1000 kcal)	54⋅1^a^	55⋅2^a^	48⋅2^a^	53⋅8^a^
Iron (mg/1000 kcal)	5⋅5^a^	4⋅5^b^	5⋅3^a^	5⋅4^a^
Sodium (mg/1000 kcal)	1203^a^	1165^a^	1361^b^	1399^b^
Magnesium (mg/1000 kcal)	165^a^	154^b^	146^b^	140^b,c^
Calcium (mg/1000 kcal)	456^a^	414^b^	452^a^	443^a,b^
Zinc (mg/1000 kcal)	4⋅9^a^	5⋅0^a^	4⋅5^a^	4⋅5^b^
Potassium (mg/1000 kcal)	1518^a^	1620^b^	1345^b,c^	1307^b,c^
Iodine (mg/1000 kcal)	66⋅9^a^	82⋅9^b^	62⋅1^a^	62⋅9^a^
Alcohol (g/1000 kcal)	0⋅03^a^	0⋅02^a^	0⋅12^b^	0⋅05^a^
Total sugars (g/1000 kcal)	44⋅3^a^	46⋅8^a^	46^a^	47⋅5^a^

Values expressed as median. One-way ANOVA on ranks was used.

^a,b,c,d^ Significant differences between clusters at *P* < 0⋅05.

**Fig. 5.**
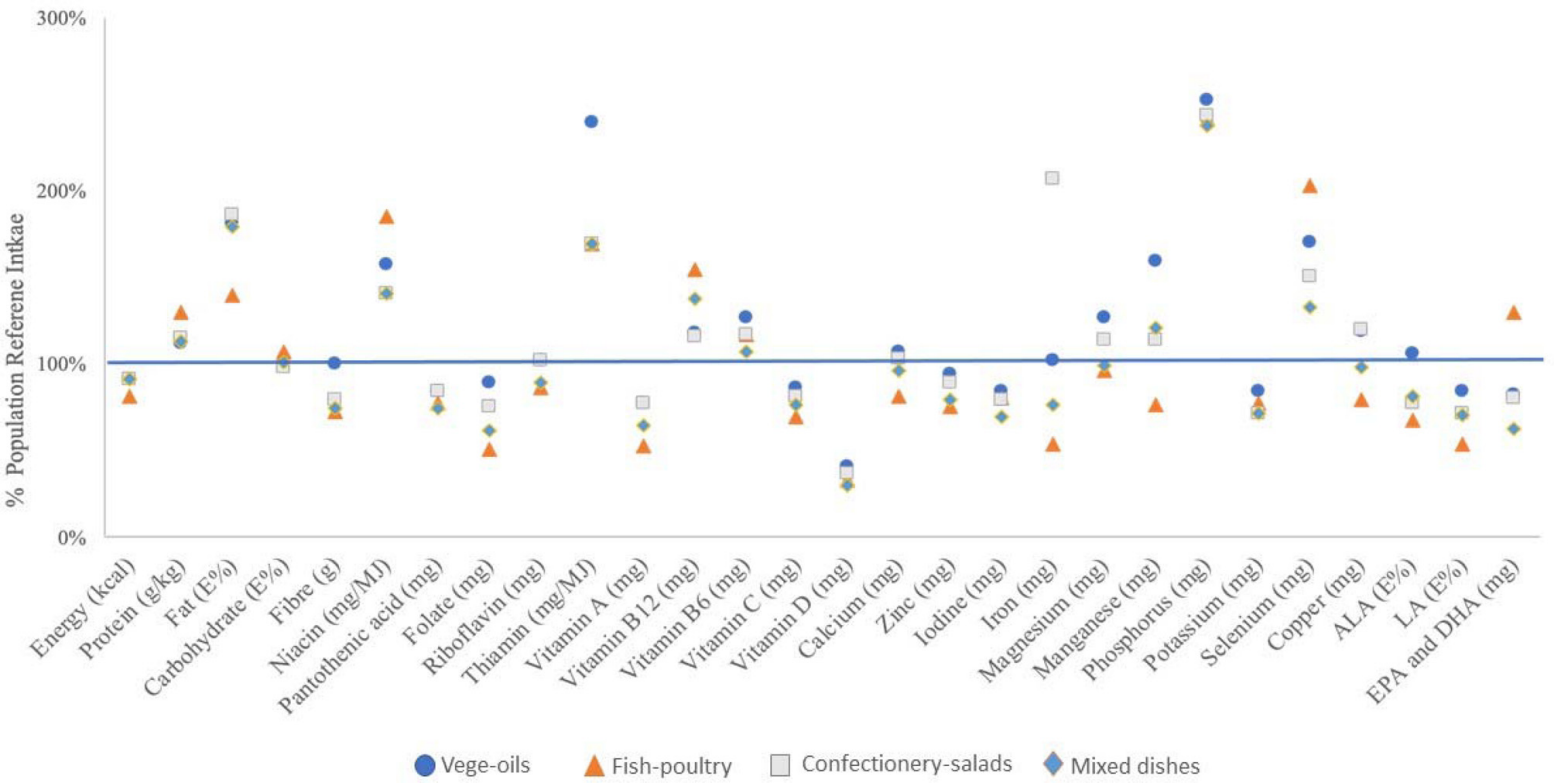
Mean nutrient intake of clusters as proportion of EFSA DRV.

The mean nutrient intakes as a proportion of EFSAs’ dietary reference values (DRVs) are depicted in Fig. 5. Except for manganese and iron, there tended to be little variation between the four clusters. Some nutrients showed a substantial deviation from the DRV; for all clusters, the consumption of energy, vitamin A, vitamin C, vitamin D, folate and iodine were consistently below the DRV, while fat (as percentage of energy E%), thiamine, selenium, phosphorus and niacin exceeded the DRV. Note, the DRV for fat represents a range, and for the purpose of this analysis, we chose 20 E% as the reference value. For other nutrients of public health concern, such as fibre and ALA, only the ‘vege-oils’ cluster met the relative DRVs. For calcium, only the ‘vege-oils’ cluster and the ‘confectionery-salads’ cluster met the DRV, while only the ‘fish-poultry’ cluster met the DRV for DHA.

Additionally, overall diet adequacy, expressed as MAR, was not significantly different between clusters. The highest MAR was found in the ‘vege-oils’ cluster (0⋅76), while the clusters ‘confectionery-salads’ and ‘mixed dishes’ showed a MAR of 0⋅72 and the ‘fish-poultry’ cluster of 0⋅70.

## Discussion

To the best of our knowledge, the present study is the first analysis that aimed to identify dietary patterns in European lactating mothers using cluster analysis. We chose cluster analysis because it separates individuals into mutually exclusive clusters of participants who consume similar foods^(15,25,26)^. The derived clusters are relatively homogenous groups in terms of food items consumed. This approach allows for a detailed comparison of energy and nutrient intakes between the clusters and assessment of nutritional adequacy for each pattern^(27,28)^. We identified four distinct dietary patterns in European lactating women. Those clusters were ‘vege-oils’, ‘fish-poultry’, ‘confectionery-salads’ and ‘mixed dishes’. These patterns reflect specific nutritional intake within clusters. Overall, protein and fat intake were meeting the European DRVs, while the intake of folate, vitamin C, vitamin A, vitamin D and iodine did not meet European DRVs.

Looking at the individual clusters in the present study, most lactating women were classified as having a ‘fish-poultry’ dietary pattern, followed by ‘mixed dishes’, ‘vege-oils’ and ‘confectionery-salads’. None of the dietary patterns met EFSA's DRVs for all nutrients, which is consistent with reports from other non-European populations. Although statistically not different, the ‘vege-oils’ cluster tended to have the highest nutritional adequacy. The Mediterranean diet, to which the ‘vege-oils’ dietary pattern can be broadly compared, is also characterised by high consumption of nuts and is generally associated with higher dietary quality scores^(29)^. The variation of nutrients consumed between clusters was usually small with the exception of iron, which was lowest in the ‘fish-poultry’ cluster. Women in this cluster tended to consume protein from food sources such as ‘fish’, ‘poultry’, ‘cereals and pasta’, ‘starchy tubers’, ‘red meat’ and ‘milk and milk product’, which are reasonable sources for haem iron. However, the ‘fish-poultry’ type food intake did not translate into sufficient amount of iron intake, which could due to the lack of consumption of iron-rich legumes and vegetables (Supplementary Table S5 of Supplementary material). It is true that the bioavailability of iron from plant-based foods is lower than that of animal-based foods, this analysis showed the quantity of iron rather than quality. On the other hand, it cannot be excluded that other factors influenced the findings for iron. While the use of supplements may be common in some European countries, only five participants in this cohort reported taking supplements. The suboptimal nutrient adequacy observed in our and other studies may be partly explained by reports that lactating women are rather resistant to adapt their dietary habits to meet their increased nutritional needs^(3,4,30,31)^.

The dietary intake of lactating women varies across studies. A survey conducted in France evaluated the food and nutritional intake of lactating women and found the average energy intake was 1669 kcal^(32)^, which is substantially lower than the 2044 kcal observed for the total population of lactating women in the present study. In the French survey, lactating women also did not meet dietary intake recommendations for several micronutrients, as was also observed in the present study. The observation of micronutrient deficiencies is a phenomenon that was confirmed for the general, male and female adult population in France where the nutrient adequacy, expressed as MAR, ranged between 69 and 86 % between seven clusters^(33)^. A recent analysis of urban lactating women in China showed a suboptimal dietary patterns^(34)^. Similar to our observations, energy was found to be below local RNI, while fat E% was above. In alignment with the present results, inadequacy in nutrient intake was observed for vitamin A, vitamin C and dietary fibre. However, the magnitude of deviation from RNI for the nutrients at risk was slightly greater in Chinese lactating women than what we observed in the European population. Yu *et al.* applied linear programming to model an optimised diet; even in the modelled diet, it was demonstrated that it would be a challenge to meet the needs for vitamin A, vitamin B1, vitamin B6, calcium, selenium and dietary fibre for lactating mothers in China^(34)^.

A study in Indonesia shown that only 57 % of lactating women were reported to have an adequate intake of micronutrients^(13)^. The discrepancies may well be rooted in access to certain food or food fortification practices which may differ between China, Indonesia and Europe. Interestingly, many European lactating mothers in the present study did not meet their folate needs either, with the mean folate intake ranging across clusters from 51 to 89 % of the DRV. The lowest folate intake was observed in the cluster ‘fish-poultry’, with about 51 % of the DRV. This is of concern, as folate is of prime interest in women of child-bearing age, particularly important for the periconceptional period.

Lactating women have greater nutritional demands than non-lactating women because of their increasing nutritional needs during lactation and ensure optimal infant growth^(31)^. The diet of lactating women is of prime interest because HM is the optimal nutrition for healthy newborn infants. The nutritional and non-nutritional composition of HM is affected by numerous factors, of which the mother's diet is a fundamental aspect. Although we did not assess the nutrient composition of HM, a mother's nutritional requirements, including energy requirements, during lactation tend to increase to meet demands of maximal mammary gland secretory capacity as well as optimal nutrient composition of the milk^(31)^. While the nutrient composition of breast milk may be associated with maternal age and BMI, maternal intake of nutrients such as polyunsaturated fatty acids (PUFA), *n*-3 fatty acids, including eicosapentaenoic acid (EPA) and DHA, *n*-6 FA and SFA is positively correlated with the corresponding FAs in the HM^(35)^. However, others found no strong correlation between dietary nutrients and breast milk components^(36)^, or suggest that it is not diet, but the maternal body composition that may be associated with the nutritional value of HM^(37)^. Scientific evidence suggests that some nutrients in HM can be modified by maternal diets, while others cannot. For example, there is evidence that calcium in HM is not impacted by maternal diet, while for several other nutrients, such as B-vitamins, there is evidence that the maternal diet influences their content levels in HM^(38,39)^.

The present study has several strengths. Importantly, this multicentre study included participants from six different European countries, representing a good reflection of cultural and geographical variability, making the present results relevant for a European population. Furthermore, for each participant, we collected the food intake of 3 d at five visits, which represents a solid foundation to calculate nutrient intake. The 3-d food diaries were supervised by trained interviewers, and the information captured directly, hence decreasing information bias. Cluster analysis is one of the most frequently used methods to assess dietary patterns. The analysis is conducted *a posteriori*, and it allowed the mean nutrient intake to be quantitatively captured into four different clusters.

The limitations of the present study include that its observational design does not allow causality to be established. Furthermore, France and Portugal had the highest number of participants in the present study, together representing about 70 % of all participants. It is known that dietary habits as well as dietary quality vary between cultures across Europe^(40,41)^ and this may have influenced the present results of the present analysis. We only included the 4 months of lactation, which is less than the recommended 6 months of exclusive breast feeding. Sample size may need to be increased to provide more information on dietary patterns among lactating women in Europe, especially to better understand country-wise differences. No measure of underreporting was included. Blood biomarkers and nutrient composition of HM were not available; hence, we lack the understanding how the observed inadequacies of mothers’ nutrient intake might translate into health deficiencies. Lastly, potential errors in assessing dietary intake cannot be ruled out.

Overall, the present study results indicated, regardless of the dietary pattern, that there is room to improve nutrient intake of lactating women to meet DRVs. Positively, the present results also provided evidence that, despite some gaps between actual intakes and DRVs, lactating women in Europe have quite diverse food consumption; hence, nutrition recommendations to improve dietary intake and to meet DRVs in this specific population should be feasible. The consequences of inadequate/adequate dietary intakes on HM composition and infant related outcomes still need to be further explored, which is the aim of ongoing research like the ATLAS project.

## Conclusion

Four dietary patterns were identified in the present analysis of European lactating mothers. Irrespective of the pattern, many nutrients were inadequately consumed when compared with the European DRVs for lactating mothers. For all clusters, protein and fat intake were above the European DRVs while folate, vitamin C, vitamin A, vitamin D and iodine were not compliant with the recommendations. A diet high in vegetables and nuts, represented in the ‘vege-oils’ cluster, tended to provide a more adequate nutrient intake in lactating women compared with the other dietary patterns, as an adequate intake for fibre and ALA was only achieved in the ‘vege-oils’ cluster and more importantly, the MAR of this cluster was the highest of all clusters.

Based on these results, nutritional recommendations for lactating women need to take the current low intake of folate, vitamin C, vitamin A, vitamin D and iodine into account. Furthermore, education should be provided to improve nutrient density by optimal food choices as well as higher adherence to healthy dietary patterns, and in cases where dietary modification alone may not be sufficient, considering fortification and supplementation. This information could provide perspectives on informing interventions that will enable the improvement of the nutritional status of lactating women. More research is required to actually link nutrient intake in lactating mothers to HM nutrient levels, infant nutrient status and growth and developmental outcomes.

## Supplementary material

For supplementary material accompanying this paper visit https://doi.org/10.1017/jns.2021.7. click here to view supplementary material
