# Supplementary material for: Analysis of dietary patterns and nutritional adequacy in lactating women: a multicentre European cohort (ATLAS study)
Source: J Nutr Sci. 2021 Mar 11;10:e17. doi: 10.1017/jns.2021.7 (PMC8057516; doi:10.1017/jns.2021.7)
Supplement: Supplementary file 1 [file S2048679021000070sup001.docx]

# Supplementary tables

Table 4 Food groups and food items according to the 2013 CIQUAL database

| **Food groups** | **Example of food items** |
| --- | --- |
| Cereals and pasta | Flours and starches, rice and other grains, pasta and semolina |
| Breads and rolls | Leavened breads, rusks and unleavened breads, sweet rolls and brioches |
| Pastry and biscuits | Cakes and pastry, biscuits, sweet, biscuits, savoury, preparations for pastries |
| Breakfast cereals and cereal bars | Not further specified |
| Milk and milk products | Milks, dietetic dairy food, cream and similar, yoghurts and similar, fromages frais, milk-based desserts, other puddings and ices, milk substitutes |
| Cheeses | Soft cheeses, blue cheeses, semi-hard cheeses, hard cheeses, processed cheeses, uncured cheeses and similar |
| Eggs and egg products | Not further specified |
| Fats and oils | Butter and other dairy fats, other animal fats, margarines and lipids of mixed origin, vegetable oils and fats |
| Red meat | Not further specified |
| Poultry | Not further specified |
| Offals | Not further specified |
| Meat products | Not further specified |
| Fish | Fish and fish products |
| Shellfish and mollusks | Shellfish and mollusks products |
| Vegetables | Not further specified |
| Legumes | Not further specified |
| Starchy tubers | Not further specified |
| Fruits | Fresh fruits, processed fruits |
| Juices and nectars | Not further specified |
| Nuts and seeds | Not further specified |
| Sugars and confectionery | Sugars, honeys, syrups, jams, marmalades, non-chocolate confectionery, chocolate and chocolate products |
| Non-alcoholic beverages | Soft drinks, water, coffee, tea, cocoa beverages, preparations for non-alcoholic beverages |
| Alcoholic beverages | Beer and other malt beverages, ciders, perries and similar drinks, wines, liqueurs & spirits, alcoholic mixed drinks |
| Mixed salads | Not further specified |
| Mixed dishes | Meat or poultry-based dishes, fish-based dishes, vegetable-based dishes, pasta or cereal-based dishes, cheese-based dishes, pizzas, crepes and savoury pies |
| Sandwiches | Not further specified |
| Soups and stocks | Soups ready to eat, stocks ready to eat, soups and stocks to rehydrate |
| Seasonings and sauces | Herbs, spices and seasonings, condiments and savoury sauces, dessert sauces |
| Products for special nutritional use | Dietary foods for special medical purposes, foods for intense muscular effort, food intended for use in energy-restricted diets, foods for infants and young children |

Table 5 Average amount of food (in grams/day) consumed per cluster

| **Food groups** | **Vege-oils** | | **Fish-poultry** | | **Confectionery-salads** | | **Mixed dishes** | |
| --- | --- | --- | --- | --- | --- | --- | --- | --- |
|  | Mean | SD | Mean | SD | Mean | SD | Mean | SD |
| Alcoholic beverages | 47.9 | 150.2 | 8.2 | 17.7 | 25.8 | 34.8 | 28.3 | 62.7 |
| Breads and rolls | 84.0 | 50.5 | 86.5 | 34.8 | 113.4 | 45.5 | 79.4 | 32.1 |
| Breakfast cereals and cereal bars | 34.2 | 78.7 | 8.8 | 14.2 | 19.3 | 31.6 | 17.4 | 27.7 |
| Cereals and pasta | 84.3 | 46.0 | 121.4 | 53.2 | 77.0 | 45.4 | 72.1 | 36.0 |
| Cheeses | 36.7 | 21.9 | 12.0 | 10.5 | 46.4 | 27.5 | 28.2 | 19.1 |
| Eggs and egg products | 16.7 | 11.5 | 13.7 | 11.2 | 13.5 | 11.8 | 10.6 | 12.5 |
| Fats and oils | 20.2 | 9.7 | 9.3 | 4.6 | 12.8 | 9.5 | 10.7 | 6.7 |
| Fish | 28.2 | 25.1 | 55.9 | 28.8 | 22.1 | 20.2 | 17.0 | 15.8 |
| Fruits | 226.9 | 127.0 | 211.9 | 115.1 | 150.7 | 106.4 | 116.2 | 78.4 |
| Juices and nectars | 71.4 | 62.0 | 56.2 | 60.6 | 74.1 | 73.7 | 112.7 | 100.0 |
| Legumes | 13.5 | 17.3 | 6.3 | 9.0 | 5.5 | 8.4 | 3.2 | 6.3 |
| Meat products | 29.3 | 22.7 | 16.7 | 14.2 | 35.4 | 26.8 | 34.4 | 21.3 |
| Milk and milk products | 264.2 | 152.8 | 361.6 | 175.7 | 235.0 | 131.8 | 257.0 | 182.2 |
| Mixed dishes | 73.1 | 50.3 | 36.6 | 28.9 | 69.0 | 43.0 | 132.7 | 58.3 |
| Mixed salads | 2.2 | 5.4 | 2.7 | 9.5 | 18.8 | 22.1 | 4.1 | 7.2 |
| Non-alcoholic beverages | 610.4 | 882.4 | 709.9 | 618.7 | 734.3 | 797.0 | 611.4 | 626.7 |
| Nuts and seeds | 10.1 | 13.9 | 1.6 | 4.2 | 3.4 | 4.3 | 2.9 | 5.4 |
| Pastry and biscuits | 46.7 | 29.3 | 44.4 | 28.0 | 46.6 | 22.5 | 75.1 | 39.2 |
| Poultry | 20.4 | 19.4 | 52.0 | 31.6 | 22.6 | 18.7 | 25.0 | 23.8 |
| Red meat | 42.9 | 39.8 | 74.1 | 40.6 | 37.5 | 21.8 | 39.5 | 27.4 |
| Sandwiches | 0.9 | 3.9 | 3.6 | 6.5 | 6.6 | 12.8 | 16.4 | 19.6 |
| Seasonings and sauces | 22.4 | 19.3 | 6.0 | 6.9 | 18.2 | 16.0 | 14.4 | 9.6 |
| Shellfish and mollusks | 7.8 | 14.7 | 8.3 | 13.5 | 6.6 | 14.6 | 8.1 | 14.5 |
| Soups and stocks | 28.0 | 43.4 | 130.2 | 102.1 | 62.7 | 75.9 | 40.6 | 56.8 |
| Starchy tubers | 55.4 | 49.2 | 90.4 | 40.7 | 52.2 | 30.5 | 51.9 | 36.7 |
| Coffee, tea, cocoa beverages | 164.4 | 167.1 | 86.1 | 75.7 | 378.3 | 310.2 | 168.9 | 181.6 |
| Preparations for non-alcoholic beverages | 0.0 | 0.0 | 0.3 | 2.2 | 0.5 | 2.7 | 0.2 | 0.7 |
| Soft drinks | 45.5 | 83.5 | 58.7 | 97.1 | 60.1 | 102.6 | 124.3 | 136.4 |
| Sugars and confectionery | 20.7 | 13.1 | 9.6 | 11.0 | 42.8 | 22.8 | 17.9 | 13.5 |
| Vegetables | 206.6 | 91.2 | 71.8 | 51.1 | 121.7 | 45.3 | 97.6 | 57.2 |
